# Prognostic Impact of Pretreatment Plasma Fibrinogen in Patients with Locally Advanced Oral and Oropharyngeal Cancer

**DOI:** 10.1371/journal.pone.0158697

**Published:** 2016-06-30

**Authors:** Daniel Holzinger, Ivan Danilovic, Rudolf Seemann, Gabriela Kornek, Johannes Engelmann, Robert Pillerstorff, Simone Holawe, Amanda Psyrri, Boban M. Erovic, Gregory Farwell, Christos Perisanidis

**Affiliations:** 1 Department of Cranio-, Maxillofacial and Oral Surgery, Medical University of Vienna, Austria; 2 Department of Medicine I, Medical University of Vienna, Austria; 3 Division of Oncology, Second Department of Internal Medicine, Attikon University Hospital, Athens, Greece; 4 Department of Otolaryngology—Head and Neck Surgery, Medical University of Vienna, Austria; 5 Department of Otolaryngology-Head and Neck Surgery, University of California, Davis, Sacramento, United States of America; Taipei Medical University, TAIWAN

## Abstract

**Background:**

We aimed to determine the prognostic significance of pretreatment plasma fibrinigen in patients with oral and oropharyngeal squamous cell carcinoma (OOSCC).

**Methods:**

A cohort of 183 patients with locally advanced OOSCC receiving preoperative chemoradiotherapy was retrospectively examined. Using ROC curve analysis, a pretreatment plasma fibrinogen cutoff value of 447mg/dL was determined. The primary endpoints were overall survival and recurrence-free survival. A secondary endpoint was to determine whether pretreatment plasma fibrinogen could predict treatment response to neoadjuvant chemoradiotherapy. Cox regression models and Kaplan–Meier curves were used for survival analyses.

**Results:**

Seventy-one patients had an elevated pretreatment plasma fibrinogen (fibrinogen >447mg/dL). Patients with high fibrinogen showed significantly higher pathologic stages after neoadjuvant treatment than those with low fibrinogen (p = 0.037). In univariate analysis, elevated fibrinogen was associated with poor overall survival (p = 0.005) and recurrence-free survival (p = 0.008) Multivariate analysis revealed that elevated fibrinogen remained an independent risk factor for death (hazard ratio 1.78, 95% CI 1.09–2.90, p = 0.021) and relapse (hazard ratio 1.78, 95% CI 1.11–2.86, p = 0.016).

**Conclusion:**

Elevated pretreatment plasma fibrinogen is associated with lack of response to neoadjuvant chemoradiotherapy and reduced OS and RFS in patients with OOSCC. Thus, plasma fibrinogen may emerge as a novel prognostic indicator and a potential therapeutic target in OOSCC.

## Introduction

Oral and oropharyngeal squamous cell carcinoma (OOSCC) is the sixth leading cancer and a major cause of morbidity and mortality worldwide [1]. Despite substantial treatment advances, the overall survival of patients with OOSCC continues to hover around 50% at 5 years, primarily because patients develop locoregional recurrence and/or metastatic disease [2]. In recent years, cancer research has been concentrated on the characterization of novel markers, which ideally should have the potential to select patients who will benefit from specific treatment and identify those at high risk for disease recurrence and death [3].

There is strong evidence suggesting that plasma fibrinogen, an acute phase glycoprotein that is associated with the maintenance of hemostasis, is a central factor in both inflammation and cancer development [4]. The results of numerous clinical studies have shown that elevated pretreatment plasma fibrinogen levels are associated with worse survival in a diversity of malignancies, including lung, gastroesophageal, colorectal, ovarian, pancreatic, and hepatobiliary cancer [5]. Evidence for the use of plasma fibrinogen as predictor of clinical outcome in patients with head and neck cancer is limited [6–8]. Given this background, the purpose of this study was to assess the value of pretreatment plasma fibrinogen in predicting overall survival (OS) and recurrence-free survival (RFS) in patients with locally advanced OOSCC who received preoperative chemoradiotherapy. We hypothesized that elevated pretreatment plasma fibrinogen represents a marker of worse survival in patients with oral and oropharyngeal cancer.

## Patients and Methods

### Study Population and Treatment

The study population comprised patients with primary locally advanced OOSCC who were treated with curative-intent neoadjuvant chemoradiation (CRT) followed by radical cancer surgery at the Departments of Radiotherapy and Cranio-Maxillofacial and Oral Surgery, at the Medical University of Vienna, between 2000 and 2011. Patients suitable for inclusion in this study had to meet the following criteria: (i) biopsy-confirmed primary OOSCC, (ii) no previous treatment for OOSCC, (iii) disease Tumour Node Metastasis (TNM) stages III and IV, (iv) World Health Organization (WHO) performance status and laboratory parameters allowing chemotherapy and surgery, (v) clear resection margins (R0), and (vi) available complete blood counts, including plasma fibrinogen, obtained up to 1 week prior to neoadjuvant chemoradiotherapy (pretreatment). At the time of sampling, no patient showed any sign of active inflammation or infection. Exclusion criteria were: (i) staging with distant metastatic disease (M1), (ii) previous history of squamous cell carcinomas of the head and neck, and (iii) coagulation disorders. All patients underwent neoadjuvant CRT consisting of mitomycin C (15 mg/m^2^, i.v. bolus injection on day 1) administered with 5-fluorouracil (750 mg/m^2^/day, continuous infusions on days 1–5) and concurrent radiotherapy over 5 weeks up to a total dose of 50 Gy (25 fractions of 2 Gy per day). The surgical procedure was scheduled 4–8 weeks after the end of radiotherapy. All patients received the same protocol of surgery consisting of radical resection of the primary tumor, guided by pretreatment margins defined by ink tattoo, with a macroscopic safe margin of at least 1 cm and concurrent neck dissection according to pretreatment lymph node status (neck dissection in levels I–III was performed for the clinically negative neck (N0), and neck dissection in levels I–V for clinically positive neck lymph nodes (N+)). Patients were followed up on a regular basis (in 3-month intervals during the first 2 years and then in 6-month intervals for the next 3 years) for a minimum of 5 years or until death.

For all eligible patients a database was generated using demographic, clinicopathological and follow-up data that were extracted from the Vienna General Hospital Patient Information System (AKIM) together with data obtained from surgical and pathologic charts. The clinical and pathological tumor staging was determined according to the TNM classification of the International Union Against Cancer (UICC). Pathological examination of the surgical specimens was performed by means of an institutional protocol providing information on histological grade (WHO classification criteria), extension of the primary tumor, lymph node status, perineural invasion, and resection margins. The local Institutional Review Board (the Ethics Committee of the Medical University of Vienna and Vienna General Hospital) approved this retrospective cohort study.

### Fibrinogen Measurement

As a part of the clinical routine, blood samples were obtained by peripheral venous puncture after overnight fasting and complete blood counts including plasma fibrinogen levels were measured 24 hours to 1 week prior to neoadjuvant treatment. Plasma fibrinogen was assessed according to the Clauss method using clotting reagents from Diagnostica Stago (Asnieres sur Seine, France) [9].

### Statistical Methods

The primary endpoints of this study were: (i) OS defined as the time from surgery to death from any cause or last follow-up, and (ii) RFS defined as the time from surgery to disease recurrence (locoregional or distant recurrence), death without recurrence or last follow-up. A secondary endpoint was to determine whether pretreatment plasma fibrinogen could predict treatment response to neoadjuvant CRT. Descriptive statistics were used to summarize the clinicopathological characteristics of the study cohort. Assessed variables were age, sex, smoking, clinical T category, clinical N category, tumor grade, post-treatment pathologic TNM stage (ypTNM), perineural invasion, as well as pretreatment plasma fibrinogen. Receiver operating characteristic (ROC) curves for OS and RFS prediction were constructed to estimate the optimal cutoff points for plasma fibrinogen. The optimal cutoff value was determined as the point on the ROC curve that maximizes the Youden Index. The area under the ROC curve (AUC) was used to calculate discrimination ability. The associations between clinicopathological variables and pretreatment plasma fibrinogen were assessed using either the chi-square test or the trend version of chi-square test, as appropriate. Survival curves were generated by means of the Kaplan–Meier method, and the log-rank test was used to evaluate survival differences between groups. Hazard ratios (HR) and 95% confidence intervals (CI) were calculated using univariate and multivariate (backward method) Cox proportional hazards regression models to analyze the effects of prognostic variables OS and RFS. Statistical significance was defined as a two-sided p-value <0.05. Statistical analysis of data was performed using the Statistical Package for the Social Sciences (SPSS®, version 21.0; IBM Corp., Armonk, NY) and MedCalc statistical software v12.2.1.0.

## Results

### Patients’ Characteristics

**Fig 1:**
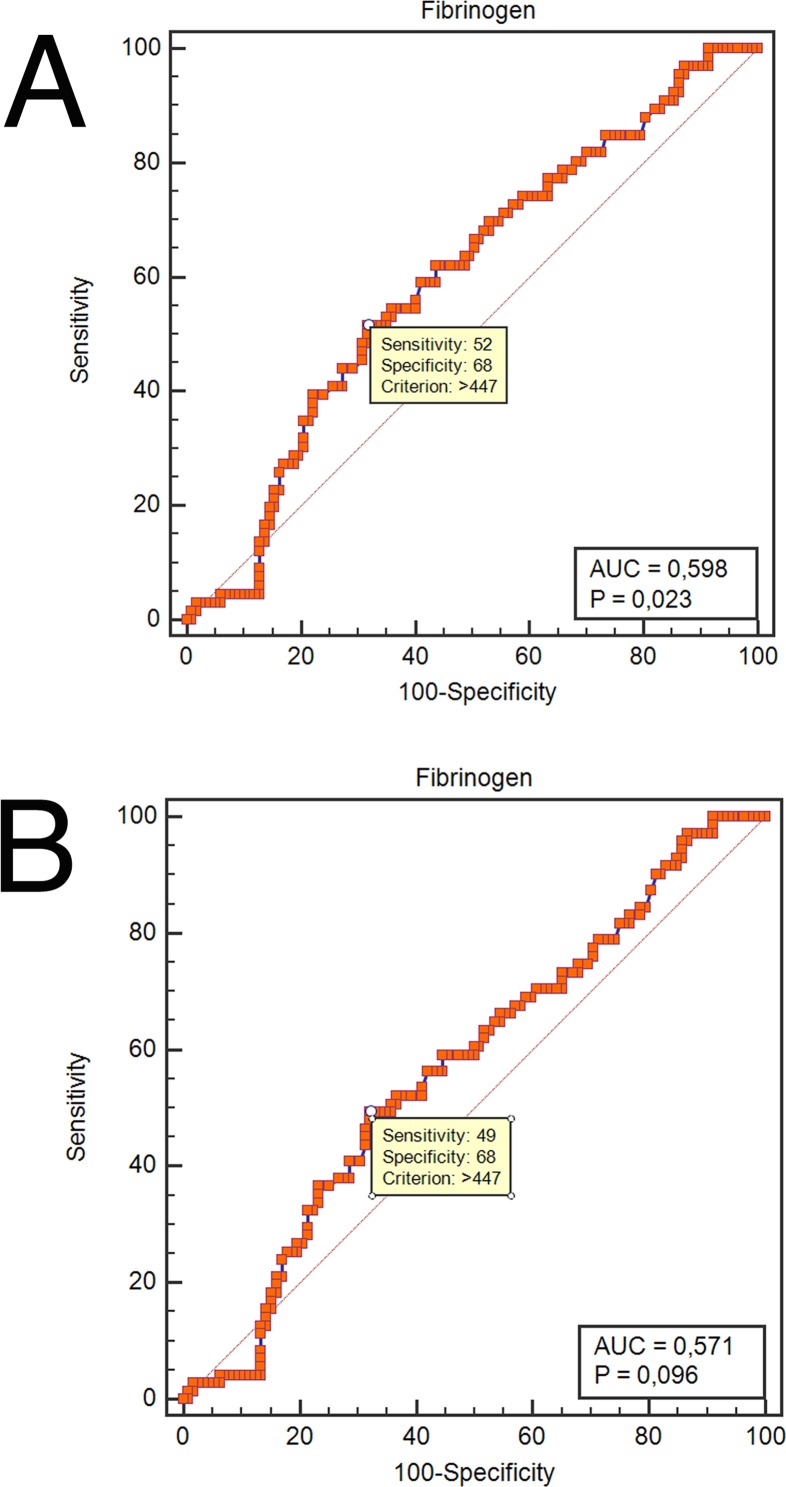
**Receiver operating characteristic curves for pretreatment plasma fibrinogen predicting overall survival (A), and recurrence-free survival (B).** Using ROC curve analysis, a pretreatment plasma fibrinogen cutoff value of 447mg/dL was determined for both OS and RFS.

Patient clinical and pathological characteristics are shown in Table 1. A total of 183 patients with locally advanced OOSCC who received neoadjuvant chemoradiation followed by surgery met the eligibility criteria for this study. The mean baseline plasma fibrinogen level was 420.6mg/dL (standard deviation ± 111.2). Using ROC curve analysis, a pretreatment plasma fibrinogen cutoff value of 447mg/dL was determined for both OS (sensitivity = 52%; specificity = 68%; corresponding AUC = 0.598; Fig 1A) and RFS (sensitivity = 49%; specificity = 68%; corresponding AUC = 0.571; Fig 1B). Fibrinogen values equal to or below the obtained cutoff point were considered low (n = 112/183 patients), while fibringen values above the cutoff point were defined as high (n = 71/183 patients).

**Table 1:** Correlation between pretreatment plasma fibrinogen levels and clinicopathological parameters in 183 patients with oral and oropharyngeal cancer.

		Pretreatment plasma fibrinogen (mg/dL)		
Variable	Patients n (%)	Low fibrinogen (≤447) n (%)	High fibrinogen (>447) n (%)	p^*a*^
Total n of patients	183 (100)	112 (61)	71 (39)	
Age				0.94
≤60 years	114 (62)	70 (62)	44 (62)	
>60 years	69 (38)	42 (38)	27 (38)	
Sex				0.17
Male	134 (73)	78 (70)	56 (79)	
Female	49 (27)	34 (30)	15 (21)	
Smoking				0.99
Current	152 (83)	93 (83)	59 (83)	
Former or never	31 (17)	19 (17)	12 (17)	
Clinical T-category				0.26
T1-T2	47 (26)	32 (29)	15 (21)	
T3-T4	136 (74)	80 (71)	56 (79)	
Clinical N-category				**0.042**
N0	31 (17)	24 (21)	7 (10)	
N+	152 (83)	88 (79)	64 (90)	
Tumor grade				**0.002**
G1	20 (11)	16 (14)	4 (5)	
G2	144 (79)	91 (81)	53 (75)	
G3	19 (10)	5 (5)	14 (20)	
ypTNM stage				**0.037**^**b**^
ypT0N0M0	91 (50)	62 (55)	29 (41)	
ypT0N+M0	14 (8)	10 (9)	4 (6)	
ypTNM I	43 (23)	22 (20)	21 (30)	
ypTNM II	6 (3)	4 (4)	2 (3)	
ypTNM III	11 (6)	6 (5)	5 (7)	
ypTNM IV	18 (10)	8 (7)	10 (14)	
Perineural invasion				0.85
No	164 (90)	100 (89)	64 (90)	
Yes	19 (10)	12 (11)	7 (10)	

^a^Chi-square test unless otherwise specified.

^b^Trend version of chi-square test.

Abbreviations: G1, well differentiated; G2, moderately differentiated; G3, poorly differentiated.

### Pretreatment Plasma Fibrinogen Predicts Response to Neoadjuvant CRT

Of 183 patients, 91 (50%) achieved pathologic complete tumor response after neoadjuvant treatment (ypT0N0M0), determined by the absence of residual invasive cancer within both the primary site and regional lymph nodes. Pretreatment plasma fibrinogen status (low vs. high) was statistically significant associated with pathologic stage after neoadjuvant CRT (Table 1). Patients with elevated fibrinogen (fibrinogen >447mg/dL) showed significantly higher pathologic stages after neoadjuvant treatment than those with low fibrinogen (fibrinogen ≤447mg/dL) (p = 0.037). Elevated plasma fibrinogen was statistically significant correlated with clinical N+ category (p = 0.042) and higher tumor grade (p = 0.002), while no association between fibrinogen and clinicopathological parameters, such as age, sex, smoking, clinical T-category, and perineural invasion was found.

### Pretreatment Plasma Fibrinogen Correlates with Overall Survival and Recurrence-Free Survival

The median follow-up of the total study population was 3.7 years. During this period, 66 of 183 patients died (34%). The estimated 2-year and 5-year overall survival rates of all 183 patients were 75.1% and 57.4%, respectively. The recurrence-free survival rate was 72.8% at 2 years and 54.5% at 5 years.

**Fig 2:**
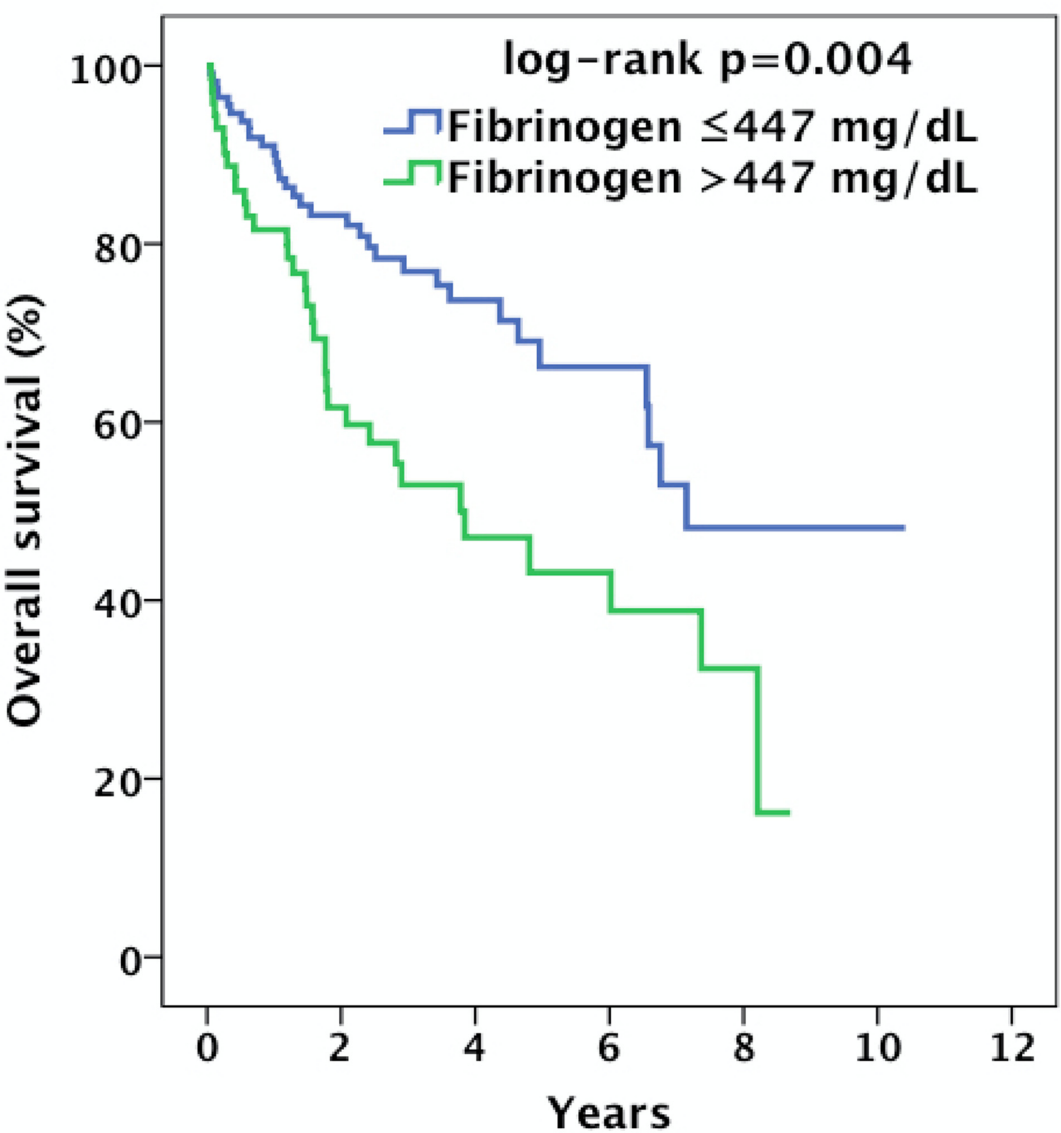
Kaplan–Meier estimates of the probability of overall survival in 183 patients with locally advanced OOSCC according to low and high pretreatment plasma fibrinogen.

**Fig 3:**
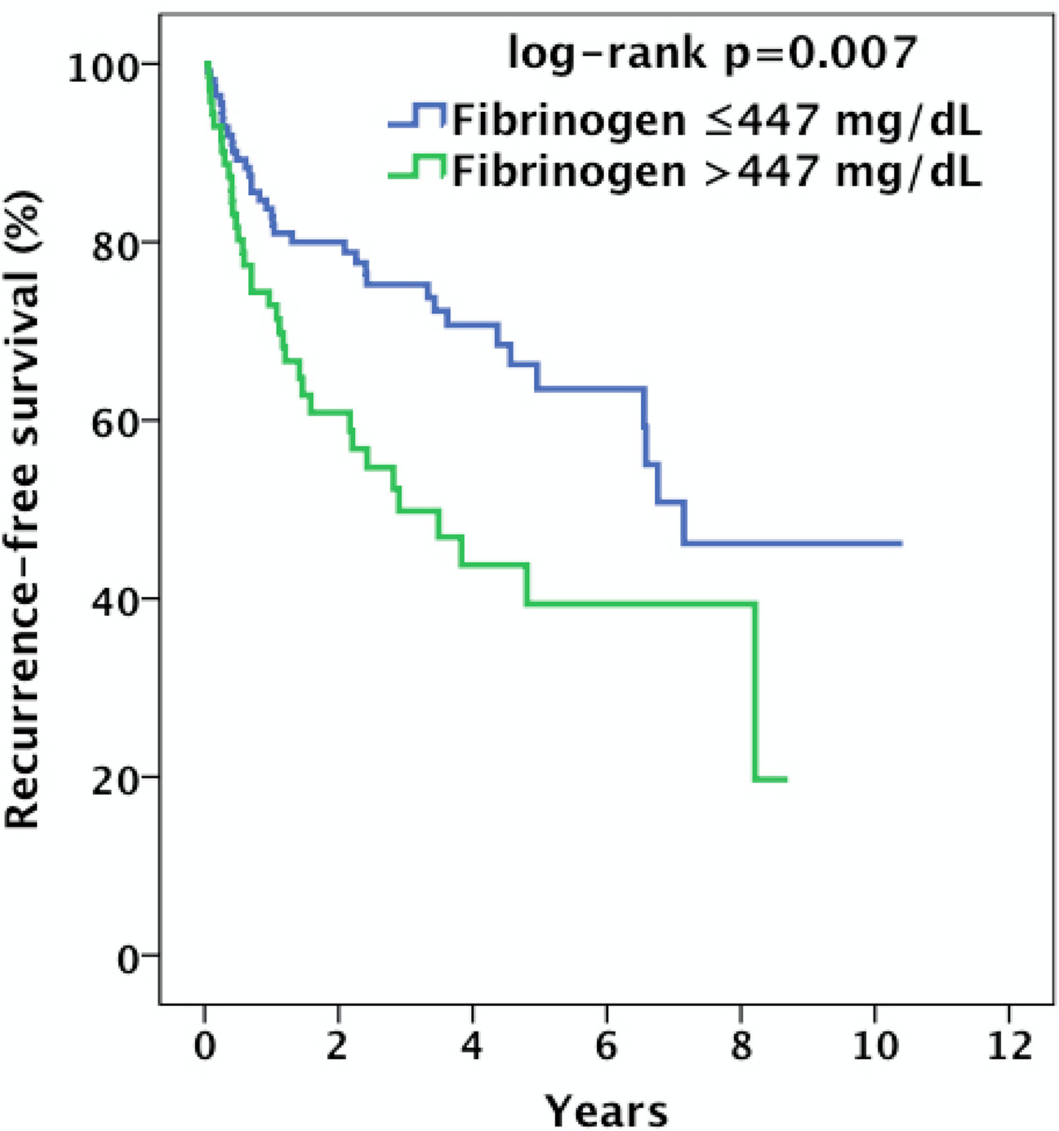
Kaplan–Meier estimates of the probability of recurrence-free survival in 183 patients with locally advanced OOSCC according to low and high pretreatment plasma fibrinogen.

Kaplan–Meier curves revealed that patients with high pretreatment plasma fibrinogen had a significantly shorter overall survival compared to patients with low fibrinogen (log-rank p = 0.004) (Fig 2). The overall survival probability at 5 years was 43.1% for patients in the high fibrinogen group and 66.2% for patients in the low fibrinogen group. Additionally, patients with high fibrinogen had a significantly shorter recurrence-free survival than those with low fibrinogen (log-rank p = 0.007) (Fig 3). The recurrence-free survival probability at 5 years was 39.4% for patients in the high fibrinogen group and 63.5% for patients in the low fibrinogen group.

Univariate and multivariate analyses of prognostic factors for OS and RFS using Cox proportional hazards regression models are presented in Table 2. In univariate analysis, patients with elevated pretreatment plasma fibrinogen experienced a significantly shorter OS than those with low fibrinigen (p = 0.005). Advanced ypTNM (p = 0.017) and positive perineural invasion (p < 0.001) were also significantly associated with worse OS. A multivariate regression model confirmed that high plasma fibrinogen was independently associated with shorter OS (HR 1.78, 95% CI 1.09–2.90, p = 0.021). In univariate analysis, elevated pretreatment plasma fibrinogen (p = 0.008), advanced ypTNM (p = 0.004) and positive perineural invasion (p < 0.001) were statistically significantly associated with worse RFS. In multivariate analysis high plasma fibrinogen (HR 1.78, 95% CI 1.11–2.86, p = 0.016) remained independently associated with poor RFS.

**Table 2:** Univariate and multivariate analysis of prognostic factors for overall survival and recurrence-free survival in patients with oral and oropharyngeal cancer.

	Overall survival						Recurrence-free survival					
	Univariate analysis^b^			Multivariate analysis^b^			Univariate analysis^b^			Multivariate analysis^b^		
Variable^a^	HR	95% CI	p	HR	95% CI	p	HR	95% CI	p	HR	95% CI	p
Age	1.15	0.69–1.92	0.58				1.15	0.71–1.88	0.56			
Sex	0.73	0.41–1.30	0.28				0.81	0.46–1.39	0.44			
Smoking	0.56	0.25–1.23	0.15				0.57	0.27–1.20	0.14			
Clinical T-category	1.01	0.59–1.71	0.98				0.98	0.58–1.64	0.95			
Clinical N-category	2.68	0.97–7.39	0.057				2.00	0.86–4.63	0.10			
Tumor grade	1.33	0.77–2.30	0.30				1.36	0.81–2.28	0.23			
ypTNM stage	0.91	0.84–0.98	**0.017**				0.89	0.83–0.96	**0.004**			
Perineural invasion	3.34	1.71–6.52	**<0.001**	3.25	1.66–6.36	**0.001**	3.18	1.68–6.02	**<0.001**	2.93	1.55–5.54	**0.001**
Baseline plasma fibrinogen	2.00	1.23–3.25	**0.005**	1.78	1.09–2.90	**0.021**	1.89	1.18–3.02	**0.008**	1.78	1.11–2.86	**0.016**

^a^Variables were coded as described in Table 1. Baseline plasma fibrinogen was coded as low fibrinogen (≤447) and high fibrinogen (>447).

^b^Cox proportional hazards regression models (backward method for multivariate analysis).

Abbreviations: OS, overall survival; RFS, recurrence-free survival; HR, hazard ratio; CI, confidence interval.

## Discussion

In this study, we analyze a cohort of patients with locally advanced OOSCC to provide evidence on the prognostic value of pretreatment plasma fibrinogen. We show that high pretreatment plasma fibrinogen is independently associated with worse long-term overall survival and recurrence-free survival in patients with OOSCC. Our study also demonstrates that patients with elevated pretreatment plasma fibrinogen have a significantly worse treatment response to neodjuvant chemradiotherapy compared to those with low fibrinogen. Taken together, our findings indicate that pretreatment plasma fibrinogen may be a novel prognostic marker in OOSCC.

The molecular mechanisms underlying the correlation between high pretreatment plasma fibrinogen and worse survival of patients with OOSCC have not been clarified yet. Nevertheless, numerous in vitro studies have shown that fibrinogen mediates cancer cell proliferation, epithelial-to-mesenchymal transition (EMT), invasion, angiogenesis, and hematogenous metastasis, and therefore plays an important role in cancer progression [10–12]. Experimental studies have demonstrated that fibrinogen binding to secreted growth factors, such as members of the transforming growth factor-b (TGF-b), platelet-derived growth factor (PDGF), vascular endothelial growth factor (VEGF), and fibroblast growth factor (FGF) families, promotes tumor cell proliferation, inhibition of apoptosis, angiogenesis, and metastasis [13]. It has been shown that tumor cells have the ability to produce endogenous fibrinogen and that FGF-2 binding to fibrinogen induces endothelial cell proliferation, thus resulting in increased angiogenesis [14, 15]. Recent research has revealed that fibrinogen acts as a bridge between integrins on platelets and circulating tumor cells (CTCs), and thus increases platelet adhesion to tumor cells [16]. In the presence of thrombin, fibrinogen converts into a compact fibrin matrix, which in combination with platelets forms a shielding cover around CTCs that protects them from natural killer cell-mediated elimination [17].

It is generally acknowledged that cross-talk exists between coagulation, the inflammatory response, and cancer progression [4, 18]. In the tumor microenvironment, fibrinogen mediates leukocyte adhesion to endothelial cells and production of the pro-inflammatory cytokines in peripheral blood mononuclear cells [19, 20]. Recent research has shown that fibrinogen-leukocyte integrin receptor aMb2 interactions induce a fibrinogen-dependent inflammatory response, which has a substantial effect on the pathogenesis and progression of cancer [10]. In our study, the observed adverse effects of elevated pretreatment plasma fibrinogen on OS and RFS of patients with OOSCC may reflect the substantial role of fibrinogen in an inflammatory tumor microenvironment that favors tumor development.

A recent systematic review and meta-analysis provided strong evidence that an elevated pretreatment plasma fibrinogen is an independent predictor of worse OS and RFS in a wide variety of malignancies, including lung, colorectal, renal, ovarian, cervical, endometrial, and head and neck cancers [5]. In accordance with this meta-analysis, our study highlights the independent prognostic effect of pretreatment plasma fibrinogen in patients with OOSCC. Our findings, if validated in larger patient populations, have widespread clinical implications. Plasma fibrinogen is a universally available, routinely measured, and inexpensive biomarker, which can be easily integrated into the clinical management of cancer patients. Furthermore, plasma fibrinogen could be simply applied in addition to established prognostic factors further improving prognosis estimation, patient consulting, and treatment decision-making in patients with OOSCC. Additionally, the evidence provided by the present study along with a convincing biological rationale associating fibrinogen with cancer progression suggest that adjunct treatments lowering plasma fibrinogen levels and/or therapies targeting fibrinogen-dependent interactions may hold promise for prolonging survival of patients with OOSCC.

This retrospective study is subject to several inherent limitations, such as the retrospective design and the relatively small sample size. Furthermore, surgery followed by radiotherapy or chemoradiotherapy is presently the mainstay of treatment for patients with locally advanced OOSCC; nonetheless, some institutions using neoadjuvant CRT followed by surgery in the management of patients with locally advanced OOSCC report an improved survival outcome [21–23]. A unique aspect of the neoadjuvant treatment concept is that it offers the opportunity to evaluate treatment response by determining pathologic stage after CRT and surgery. Moreover, the human papillomavirus (HPV) status was not considered in the group of patients with oropharyngeal cancer, which may introduce a bias in the estimated effects. However, patient groups with tumor localization either in the oropharynx or the oral cavity were similar with respect to demographic characteristics (data not shown) and survival rates (the overall survival probability at 3 years was 73% for patients with oropharyngeal cancer and 67% for patients with oral cancer; log-rank p > 0.05). In addition, it was beyond the scope of this analysis to investigate the mechanisms underlying the interaction between plasma fibrinogen, the systemic inflammatory response and cancer progression. Nevertheless, evaluation of plasma fibrinogen was achieved using clinicopathological and survival data from a homogeneous cohort of uniformly treated OOSCC patients with sufficient follow-up. The aforementioned limitations suggest that further large-scale clinical studies are warranted to confirm our findings.

## Conclusion

In conclusion, our study reveals the clinical significance of plasma fibrinogen in OOSCC. We demonstrate that an elevated pretreatment plasma fibrinogen is associated with higher pathologic tumor stages after neoadjuvant treatment and reduced OS and RFS in patients with OOSCC. Our results indicate that plasma fibrinogen may emerge as a novel prognostic marker and a potential therapeutic target in OOSCC.
